# Inducing Positive Self-Conscious Emotion (Pride) Through Feedback in Cognitive Tasks: A Pilot Study

**DOI:** 10.1192/j.eurpsy.2026.11730

**Published:** 2026-07-31

**Authors:** M. Kliková, D. U. Dudysová, A. Hrubý, M. Balajková, V. Piorecká, F. Černý, J. Štrobl, J. Hubený, J. Jonáš, J. Kopřivová, K. Janků

**Affiliations:** 1 National Institute of Mental Health, Klecany; 2 3rd Faculty of Medicine; 3 Faculty of Arts, Charles University, Prague; 4 Faculty of Biomedical Engineering, Czech Technical University in Prague, Kladno, Czech Republic

## Abstract

**Introduction:**

Emotions are integral to everyday functioning, with positive self-related emotions—such as pride—playing a protective role across various domains of life. These emotions contribute to the development of enduring personal resources, which may be leveraged in psychotherapeutic contexts.

**Objectives:**

This pilot study aimed to validate a novel experimental design for inducing the self-conscious emotion of pride using randomly assigned positive and neutral feedback during repeated cognitive tasks, with the goal to establish a protocol suitable for EEG-based research.

**Methods:**

Twelve healthy adult participants completed repeated cognitive tasks— the Stroop Test and the Eriksen Flanker Task—each paired with 10 neutral and 10 positive feedback trials. Emotional responses were assessed using the Self-Assessment Manikin (SAM) for valence, intensity, and arousal; the Authentic and Hubristic Pride Scale (AHPS) to differentiate pride subtypes; and the Qualitative Assessment of Emotions (QAE) to evaluate a range of emotional states. EEG data were recorded from 11 participants using a 64-channel system during task performance, followed by microstate analysis to explore neural correlates of induced emotional states.

**Results:**

According to SAM, no statistically significant differences were found between positive and neutral feedback conditions in emotional valence (W = 7.00, p = 0.071, r = –0.611; Mdn_pos = 6.60, Mdn_neu = 6.21), intensity (W = 14.50, p = 0.372, r = –0.356; Mdn_pos = 5.00, Mdn_neu = 5.00), or arousal (W = 16.00, p = 0.834, r = –0.111; Mdn_pos = 6.30, Mdn_neu = 6.00). However, authentic pride (Mdn = 3.00) was significantly more induced than hubristic pride (Mdn = 1.14; W = 66.0, p = 0.004). According to the QAE, positive emotions were experienced with significantly greater intensity than negative emotions (W = 77.0, p = 0.003). EEG microstate analysis revealed no significant differences between feedback conditions.

**Conclusions:**

This pilot study demonstrates that the experimental design is feasible for EEG-based investigations of self-conscious emotions. While positive feedback showed a trend toward enhancing emotional valence, the effect did not reach statistical significance. The paradigm successfully elicited authentic pride more than hubristic pride, and positive emotions were experienced more intensely than negative ones. No EEG microstate differences were observed. Given the small sample size, further research is needed.

**Disclosure of Interest:**

None Declared

